# Flexible Cooperation Between Peroxisomes and the Endoplasmic Reticulum During Lipid Synthesis of *Dictyostelium*

**DOI:** 10.3390/cells15111025

**Published:** 2026-06-02

**Authors:** Dina Sofia da Silva Telinhos, Markus Maniak

**Affiliations:** Zellbiologie, Universität Kassel, Heinrich-Plett-Str. 40, 34132 Kassel, Germany; sofia.telinhos@uni-kassel.de

**Keywords:** *Dictyostelium*, lipid metabolism, ether lipid biosynthesis, ester lipid biosynthesis, endoplasmic reticulum, peroxisome, ADHAPR enzyme

## Abstract

Ether lipids in varying amounts are membrane constituents and storage material in the protist and animal kingdoms, but are largely absent from fungi and plants. Their biosynthesis pathway starts in the peroxisome and involves a set of well-conserved enzymes. Only one step, the reduction of alkyl-dihydroxyacetone-phosphate to alkyl-glycerol-3-phosphate, is mediated by so-called short-chain dehydrogenases/reductases, which are members of huge protein families. Here, using GFP fusions, we identify a peroxisomal enzyme in *Dictyostelium*, as well as a highly related protein residing in the endoplasmic reticulum. Single-gene knockouts indicate that these enzymes largely compensate for one another, suggesting a flexible redistribution of lipid metabolites between these organelles. The double knockout, however, is severely affected in ether lipid composition and displays a clear growth retardation. The defects can also be reverted by expression of the cognate yeast enzyme, demonstrating conservation of this metabolic step across kingdoms of life.

## 1. Introduction

Lipids are the macromolecules that confer individuality to the cell by separating the vital cellular functions from the exterior world. They also define organelles in the cytoplasm by providing membrane-separated aqueous spaces for specific metabolic reactions. From a chemical viewpoint, it is mostly the amphipathic character of fatty acids, as the main building blocks of lipids, that mediates separation and allows for the self-organization into biological membranes.

Fatty acids can be assembled on three molecular platforms, namely on glycerol to make ester-type phospholipids; on dihydroxyacetone-phosphate (DHAP), resulting mostly in ether-type phospholipids; and on serine to generate sphingolipids. Interestingly, all three types can be used to add another fatty acid to the remaining hydroxyl group after dephosphorylation, making triacylglycerol (TAG) [1], monoalkyl-diacyl-glycerol (MDG) [2], and 1-O-acylceramide, respectively. The former two are stored in lipid droplets (LDs) as a source to regenerate the membrane lipid pool, while 1-O-acylceramide contributes to the water barrier in mammalian skin [3].

Not too surprisingly, alterations in lipid homeostasis are associated with many diseases, mostly affecting the heart, liver, pancreas, and brain, as many sources reiterate every year [4,5,6], but a true understanding would be lacking if the basic metabolic pathways of lipid synthesis had not been worked out in detail earlier [7]. Nonetheless, common diseases of modern societies receive more interest than, e.g., rhizomelic chondrodysplasia punctata, a devastating inherited disorder, where synthesis of ether-type lipids plays a major role [8,9].

Because things can occasionally be learned even from “primitive” model systems like *Dictyostelium*, we have started to elucidate components of a rather conventional pathway leading to TAG, with FcsA activating the fatty acids originating from the diet by coenzyme A [10], GPAT linking them to glycerol-3-phosphate [11], Lpin2 dephosphorylating the phosphatidic acid molecule [12], and DGAT1 and 2 adding the third fatty acid, again via an ester bond [13].

Interestingly, an analysis of *Dictyostelium* total cell phospholipids has recently revealed that the content of ether-type phospholipids may be as high as 70% [14], much more than anticipated from earlier studies [15], putting this evolutionarily ancient organism closer to the animal line in terms of membrane lipid composition than to plants and fungi, which both apparently lack ether lipids [16].

Ether lipid synthesis is typically initiated in the peroxisome by the enzyme dihydroxyacetone-phosphate acyl-transferase (DHAPAT), transferring an acyl chain to the sn1- hydroxyl of DHAP. Next, the fatty acid is replaced by a fatty alcohol, which originates from a fatty acid reductase (FAR), through the enzyme alkylglycerone-phosphate synthase (AGPS) [17]. While these enzymes are separate entities in mammals, FAR and DHAPAT are linked into a single polypeptide chain named FARAT in protists [11,18]. Importantly, *Dictyostelium* AGPS was the first enzyme of its kind ever to be crystallized, so that the molecular mechanism of alkyl-DHAP synthesis could be derived [19]. Because alkyl-DHAP carries a carbonyl oxygen at the sn2 position, which is not suitable for linking the fatty acid, a reduction has to take place, yielding a hydroxyl group instead. It is the aim of the present work to identify this reductase forming alkylglycerol-3 phosphate.

Alkylglycerol-3 phosphate is then converted to monoalkyl-monoacyl-glycerol phosphate, whereupon it can be processed by the enzymes of glycerol lipid synthesis, i.e., it is dephosphorylated by LPIN2 [12], and receives another fatty acid by DGAT1 (but is not a substrate for DGAT2 [13]), ending as the storage lipid monoalkyl-diacyl-glycerol (MDG).

## 2. Materials and Methods

### 2.1. Cell Culture

*Dictyostelium discoideum* strain AX2 (wild-type) and mutant derivatives were used throughout this study. Cells were cultivated in HL5+ medium (Formedium, Swaffham, UK) at 21 °C under continuous light with shaking at 180 rpm. Neutral lipid formation was induced by a 3 h supplementation with palmitic acid (final concentration 200 µM) as described previously in [20].

### 2.2. Bioinformatic Analyses

DNA and protein sequences of the candidate alkyl-dihydroxyacetone-phosphate reductase (ADHAPR) genes were obtained from DictyBase (https://dictybase.dev/stockcenter, accessed on 12 February 2026). Transmembrane domains were predicted using TMHMM 2.0 (https://services.healthtech.dtu.dk/services/TMHMM-2.0/, accessed on 12 February 2026), and UniProt annotations (https://www.uniprot.org/, accessed on 12 February 2026) were used. Peroxisomal targeting signals and additional localization motifs were analyzed using PSORT II (https://psort.hgc.jp/form2.html, accessed on 12 February 2026).

### 2.3. Plasmid Construction and Gene Expression

Candidate genes were amplified either from reverse-transcribed total RNA (*DDB0214951*) or genomic DNA (*DDB0302651* and *DDB0302650*). For extrachromosomal expression, coding sequences were cloned into pDM317 or pDM323 vectors to generate N- or C-terminal GFP fusions, respectively [21]. Otherwise, pDneo2a-based integrating vectors were used where indicated [11,22].

Constructs were generated with or without endogenous stop codons to enable the appropriate GFP fusion orientation. All intermediate cloning steps were verified by sequencing prior to final vector assembly. Detailed cloning strategies, primer sequences, and plasmid identifiers are provided in Supplementary Tables S1 and S2.

### 2.4. Gene Disruption and Generation of Knockout Strains

Gene knockouts were generated by homologous recombination using blasticidin resistance (BS^r^) cassettes inserted into coding regions. Linearized knockout constructs were introduced into *D. discoideum* by electroporation. Correct integration was confirmed by diagnostic PCR using locus-specific primers.

For the generation of double knockout strains, we employed the Cre/loxP recombination system [23,24]. Following Cre-mediated excision of the BS^r^ cassette via transient expression of plasmid #1001, clones were screened for restored blasticidin sensitivity before subsequent gene disruptions were introduced. Knockout strains were verified by PCR, including confirmation of residual loxP sites. PCR was in part performed as a multiplex reaction to obtain an internal control fragment, including in the AX2 wild-type control, yielding a 499 bp product. The primers 1182 and 1416 target the gene of short-chain dehydrogenase DDB0305607.

### 2.5. Lipid Extraction and Analysis

Lipids were extracted from cells incubated for 3 h with 200 µM of palmitic acid in growth medium according to the method of [25], adapted as described previously in [20]. Neutral lipids from three independent experiments were separated by thin-layer chromatography (TLC) on high-performance TLC (HPTLC) plates with a silica gel 60 Nano-ADAMANT surface (Macherey-Nagel, Düren, Germany) using a two-step solvent system. After the first solvent front (hexane: diethyl ether: acetic acid, 80:20:1, *v*/*v*/*v*) reached 7 cm of the plate height (10 cm), the plate was air-dried and further exposed to a second solvent system (hexane: diethyl ether, 49:1, *v*/*v*) until completion. To visualize lipids, TLC plates were immersed for a few seconds in copper sulphate solution (0.6 M in 8.5% (*v*/*v*) phosphoric acid) and heated to 160 °C for 15 min to develop a signal through charring of the lipids.

Phospholipids were separated as described by [26]. Briefly, a solvent mixture of methyl acetate: n-propanol: chloroform: methanol: 0.25% KCl (*w*/*v*) in H_2_O (25:25:25:10:9, *v*/*v*/*v*/*v*/*v*) was used to separate phospholipids across the full height of the HPTLC plate. Detection was performed as described above, and lipids used as standards are described in [11]. TLC plates were digitized on an HP Scanjet 4850 or Epson Perfection V9 scanner. Densitometric analysis was performed using the “Gel Analyzer” functions in ImageJ 1.5i. In the resulting density profile, a baseline for the background is drawn. The peak area above baseline, representing the signal, is quantified and transferred to Excel. Lipid levels were first normalized to the internal extraction control and loading marker methyl oleate. Then, each lipid level in the fatty acid-fed wild-type AX2 sample was set to 1. Error bars reflect standard deviation, and significance was tested with two-tailed Student’s *t*-tests.

### 2.6. Immunofluorescence and Microscopy

Immunofluorescence staining and GFP fluorescence microscopy were performed as described previously in [27]. The endoplasmic reticulum was visualized using a mouse monoclonal antibody against protein disulfide isomerase (PDI, MAb 221-64-1) [28], and mitochondria were detected using an antibody (70-100-1) directed against *Dictyostelium* porin [29], both followed by polyclonal secondary antibody Cy3 Affinipure™ Rabbit Anti-Mouse IgG (111-165-003, Jackson ImmunoResearch Laboratories, Ely, UK). Peroxisomes were marked by co-expression of RFP-SKL [30]. Images were acquired as single confocal planes using a Leica TCS-SP laser scanning microscope or using a wide-field Leica DM5500 B fluorescence microscope (Leica Microsystems, Wetzlar, Germany).

## 3. Results

**Figure 1:**
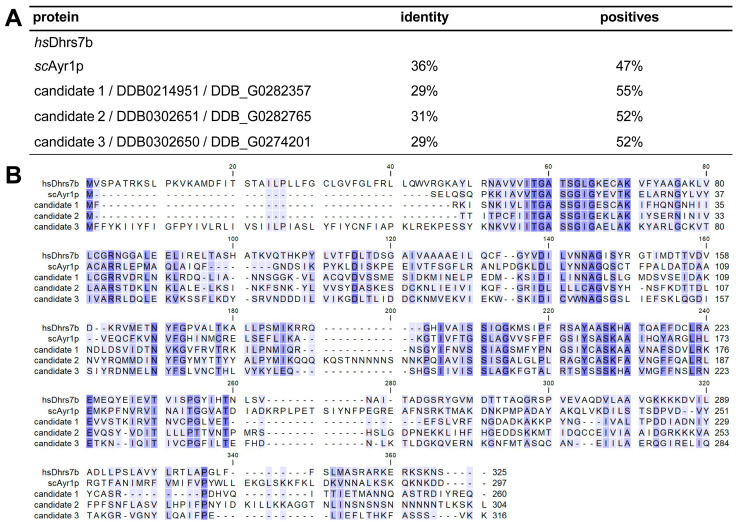
*Dictyostelium* has several ADHAPR-related proteins. (**A**) Relationship between three possible ADHAPR candidates from the *Dictyostelium* proteome with human Dhrs7b (set to 100%), showing percentages of identical residues or conservative exchanges (positives). For comparison, the first discovered ADHAPR, yeast Ayr1p (scAyr1p), is also included. (**B**) Sequence alignment of these proteins with coloured residues from dark to light blue/white according to decreasing conservation. Please note the N-terminal extensions of Dhrs7b and candidate 3, as well as the C-terminal SKL tripeptide of candidate 2, which mediate subcellular targeting (see below).

The mammalian alkyl-dihydroxyacetone-phosphate reductase (ADHAPR, EC 1.1.1.101) enzyme was biochemically characterized in the 1970s [31,32], but it took several decades to first assign the yeast gene *ayr1* to this activity [33]. Using the yeast sequence, Dhrs7b was finally identified as the mammalian ADHAPR [34]. Together, these proteins belong to a large gene family of so-called short-chain dehydrogenases/reductases (SDRs), in which sequence identities rarely exceed 30% [35]. Because *Dictyostelium* protein sequences are known to be more similar to mammals than to yeast [36], we initiated a BLAST search (V2.27.0) with human Dhrs7b against the *Dictyostelium* proteome. This search revealed a set of 43 unique short-chain dehydrogenases/reductases, which may represent potential homologues of ADHAPR. From these hits, the three top-scoring ones (Figure 1A) named here as candidate 1 (c1, DDB0214951), candidate 2 (c2, DDB0302651), and candidate 3 (c3, DDB0302650) were investigated further. A sequence alignment is shown in Figure 1B.

**Figure 2:**
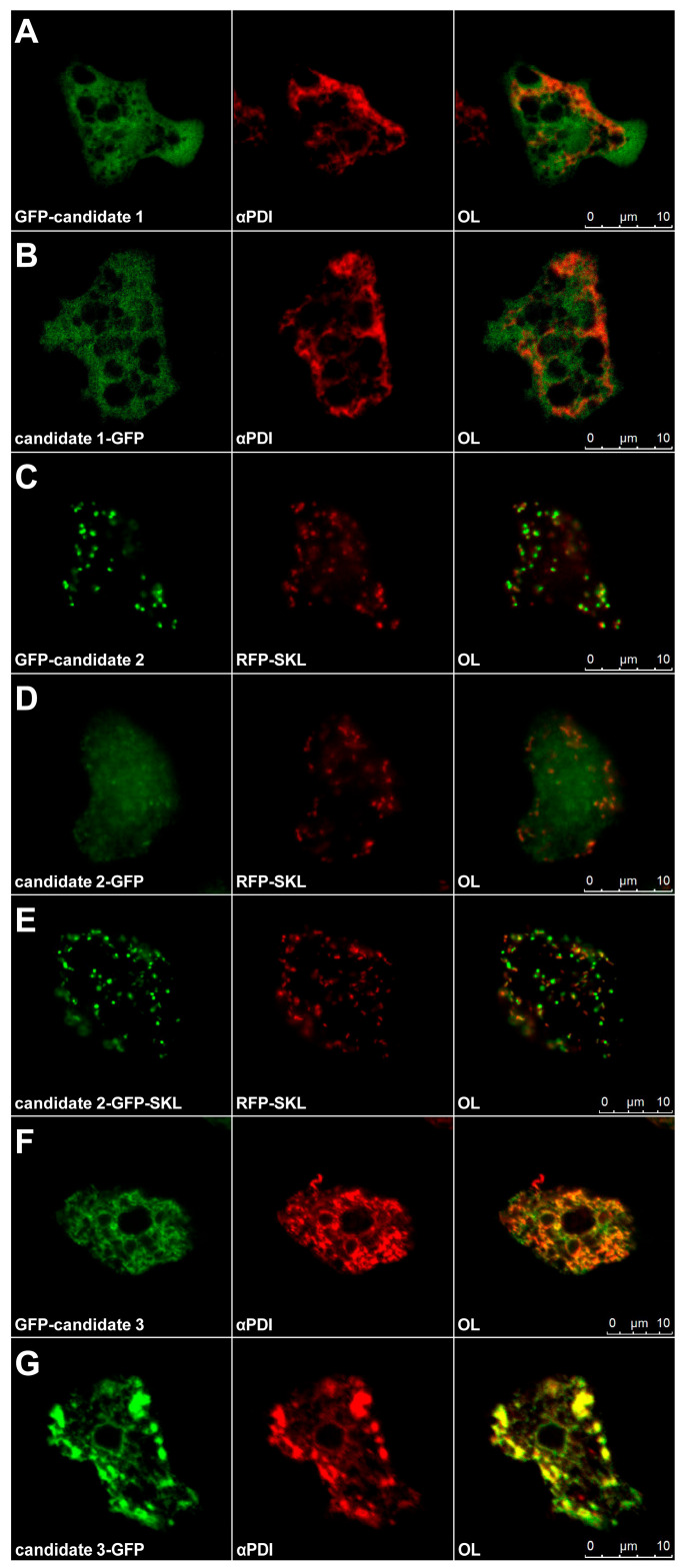
ADHAPR candidates differ in their localization. Immunofluorescence analysis from fixed wild-type cells expressing candidates 1 to 3 carrying GFP (green) at their N-terminus (made with (**A**) plasmid #1414, (**C**) plasmid #1547, (**F**) plasmid #1519) or C-terminus (made with (**B**) plasmid #1415, (**D**) plasmid #1545, (**G**) plasmid #1520). The strain in panel (**E**) was transformed with plasmid #1546 to produce a C-terminally positioned GFP that is extended by three amino acids (SKL) to provide peroxisomal targeting. For details on plasmids, see Table S1. In panels (**A**,**B**,**F**,**G**), the endoplasmic reticulum was revealed by immunofluorescence using an antibody directed against the protein disulfide isomerase (PDI, red), whereas cells shown in panels (**C**–**E**) co-express RFP-SKL, labelling peroxisomes in red. Overlay images (OL) in the third column include a 10 µm scale bar.

All candidates were analyzed for their localization using GFP fusion proteins, where the GFP was fused to either side of the protein. The highest-scoring protein, candidate 1, was found in the cytoplasm in both fusion variants (Figure 2A,B). When GFP was linked to the N-terminus of candidate 2, the signal was co-localized with peroxisomes (Figure 2C) due to a peroxisomal targeting sequence type 1 residing in the C-terminus. When this signal was blocked by GFP, the resulting hybrid protein mainly distributed throughout the cytoplasm (Figure 2D). Thus, we generated another construct that fuses GFP-SKL to the C-terminus of candidate 2. This construct was also detected in peroxisomes (Figure 2E), indicating that the remaining sequences within candidate 2 did not contain additional strong targeting information. Both fusions of GFP to candidate 3 resulted in a staining of the endoplasmic reticulum (Figure 2F,G) with GFP linked to the C-terminus showing a strong propensity for forming aggregates in the periphery of the cell (Figure 2G). Because Dhrs7b is a dual-localizing protein within the endoplasmic reticulum (ER) and peroxisomes of rodents and also in HeLa cells [37,38], we concluded that candidates 2 and 3 would be more likely to fulfil the ADHAPR function in *Dictyostelium* than candidate 1.

**Figure 3:**
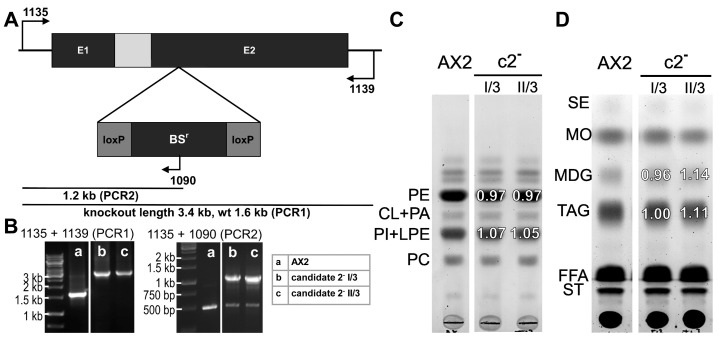
A candidate 2 mutant shows no alteration in lipid patterns and quantities. (**A**) Schematic of the genomic *c2* locus after insertion of a blasticidin S resistance (BS^r^) cassette by homologous recombination at the beginning of exon 2 (E2) using plasmid #1295. Arrows indicate diagnostic primer-binding sites outside the coding region used for targeting (1135 and 1139) and the resistance gene (1090). The positions and sizes (kb) of expected PCR products (1 and 2) are indicated beneath. For details on plasmids and primers, see Tables S1 and S2, respectively. (**B**) PCR products from genomic DNA isolated from the wild-type strain (AX2) and two independently derived *c2^-^* mutants (I/3 and II/3). Two primers situated outside the *c2* coding region (1135 and 1139) amplify a 1.6 kb fragment from wild-type DNA (a), which increases to 3.4 kb in the mutant strains (b, c), consistent with BS^r^ cassette insertion. Combining one primer binding 5′ upstream of the *c2* coding region (1135) and one primer specific for the resistance cassette (1090), the disrupted copy of the *c2* gene can only be amplified in the mutants but not in the wild type. The band at 500 bp appearing in all lanes of PCR2 is a multiplex amplification control described in Section 2. (**C**) Analysis of phospholipids in *c2* knockout strains (I/3 and II/3) by thin-layer chromatography (TLC) was performed in triplicate for quantification, and the mean values of PE and PI are expressed as fractions (white numbers) of the wild type (AX2). The results of quantifications by densitometry are provided as bar diagrams in the Supplementary Materials. One representative TLC plate is shown in the background. The positions of phosphatidylethanolamine (PE), comigrating cardiolipin (CL) and phosphatidic acid (PA), and phosphatidylinositol (PI) and comigrating lyso-PE (LPE), as well as phosphatidylcholine (PC), are shown on the left. (**D**) TLC resolving steryl esters (SEs), monoalkyl-diacyl-glycerol (MDG), triacylglycerol (TAG), free fatty acids (FFAs), and sterol (ST), as well as methyl-oleate (MO) used as a loading control, displaying the average of n = 3 experimental quantifications in relation to AX2 = 1.00.

Because the synthesis of ether lipids is initiated in the peroxisome (see Section 1), we disrupted the gene for peroxisomal candidate 2 by inserting a blasticidin resistance (BS^r^) cassette (Figure 3A) and documented the successful event by PCR analysis (Figure 3B). The phospholipid pattern from two independent knockout clones did not differ from that of the wild-type strain (Figure 3C). While fatty acid addition to the culture medium leaves phospholipids unchanged, this condition allows storage lipids to be analyzed. The neutral lipid composition of the *c2* knockouts was, however, indistinguishable from that of wild-type cells (Figure 3D).

**Figure 4:**
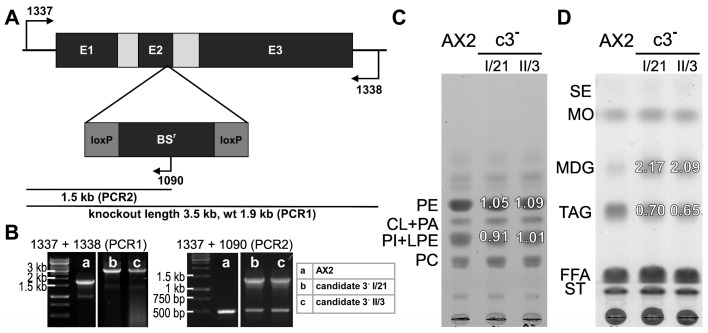
A candidate 3 knockout overproduces MDG at the expense of TAG. (**A**) Graphic representation of the *c3* gene after homologous recombination with linearized plasmid #1474 and disruption by a BS^r^ cassette in exon 2 (E2). Positioning of primers and sizes of diagnostic PCR fragments as described in Figure 3A. (**B**) PCR analysis (1) of the integration event in *c3^-^* mutants (I/21 and II/3) reveals that a wild-type (AX2) fragment of 1.9 kb (generated by primers 1337 and 1338) is shifted to 3.5 kb after insertion of the BS^r^ cassette. A 1.5 kb product in PCR2 using primers 1090 (within the resistance gene) and 1337 confirms the presence of the BS^r^ cassette. A band at 500 bp proves that the AX2 lane contains amplifiable DNA. (**C**) Phospholipids and (**D**) neutral lipids were analyzed as explained in the legend to Figure 3.

Inactivation of the candidate 3 gene followed the same rationale (Figure 4A) as before, and the documentation of the altered genetic configuration is displayed in Figure 4B. While the phospholipids appeared wild-type-like (Figure 4C), the amount of TAG as the main storage lipid was reduced by about 30%, but the content of MDG was increased over 2-fold. At first sight, this observation seems to contradict what is expected when a protein involved in the synthesis of ether lipids is inactivated. However, an alternative explanation would be that *D. discoideum* possesses a second protein with ADHAPR-like activity, for which the substrate availability is increased in the absence of candidate 3.

**Figure 5:**
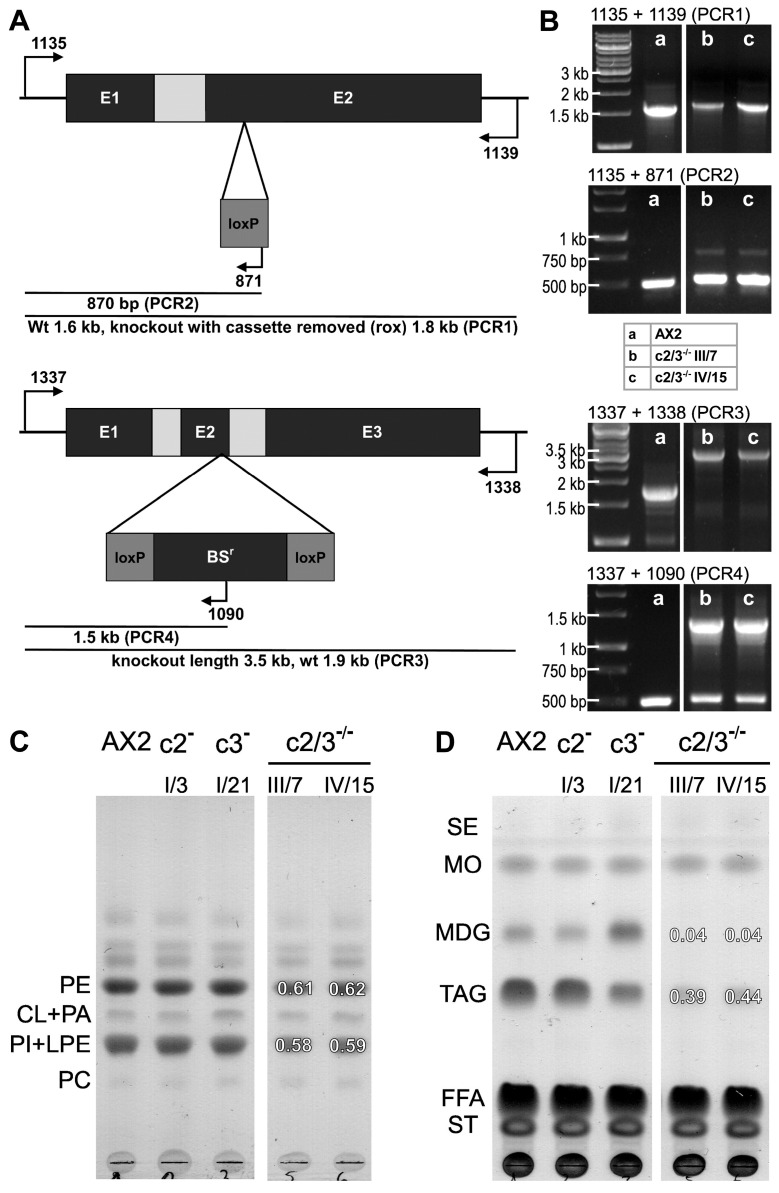
A combined loss of candidates 2 and 3 severely impairs ether lipid synthesis. (**A**) Sketch of the *c2* gene after excision of the BS^r^ cassette by the Cre recombinase (encoded by plasmid #1001), leaving a single lox P site, which still inactivates the *c2* gene by a series of stop codons in all reading frames. This genotype served as a recipient for the secondary disruption of *c3* by plasmid #1474, similar to the strategy presented in Figure 4A. (**B**) The primer combination known from Figure 3B now demonstrates the successful removal of the BS^r^ cassette from *c2* and the remaining 0.2 kb size increase (PCR1) signifies the presence of a single loxP site. A supporting result is generated by using primer 1135 together with 871 (binding within the loxP site) in PCR2. PCRs 3 and 4 utilize the same primers used previously (Figure 4B) to confirm the insertion of the BS^r^ selective marker into the *c3* locus of a *c2*^-^ mutant that occurred in clones III/7 and IV/15, verifying them as *c2/3* double knockouts. (**C**) PE and PI phospholipid species are markedly reduced in *c2/3*^-/-^ cells. (**D**) The ether-type neutral lipid MDG is virtually absent from *c2/3*^-/-^, while TAG levels have fallen to about 40% of wild-type AX2. Lipids were analyzed as explained in the legend to Figure 3.

Because, by amino acid sequence comparison, candidate 2 is the most similar protein to candidate 3 (Figure 1B), a double knockout strain lacking both candidates was generated. To this end, clone I/3 from the candidate 2 knockout was used as the base, from which the resistance cassette was first eliminated by the Cre/lox system, followed by the introduction of a secondary disruption in the candidate 3 gene (Figure 5A). From the resulting double mutant *c2/3^-/-^* clones, III/7 and IV/15 were verified to carry the correct integration by PCR (Figure 5B). It became immediately apparent that these strains grew rather slowly, their doubling times in shaken suspensions approaching 16.4 ± 0.94 (SD) hrs for strain III/7 and 16.04 ± 0.17 (SD) hrs for strain IV/15 instead of 12.1 ± 0.21 (SD) hrs for the wild type (*p* < 0.05 or *p* < 0.0001 respectively, two-tailed Student’s *t*-test, all three strains n = 3). Phospholipid analysis revealed a strong reduction in the bands representing PE and PI (Figure 5C), being the lipid species that are mainly found in the ether variant [14]. The ether-type storage lipid MDG is almost undetectable in the double knockout strains, and the TAG levels are further reduced to about 40% of the wild type (Figure 5D), suggesting a redundancy of candidates 2 and 3.

**Figure 6:**
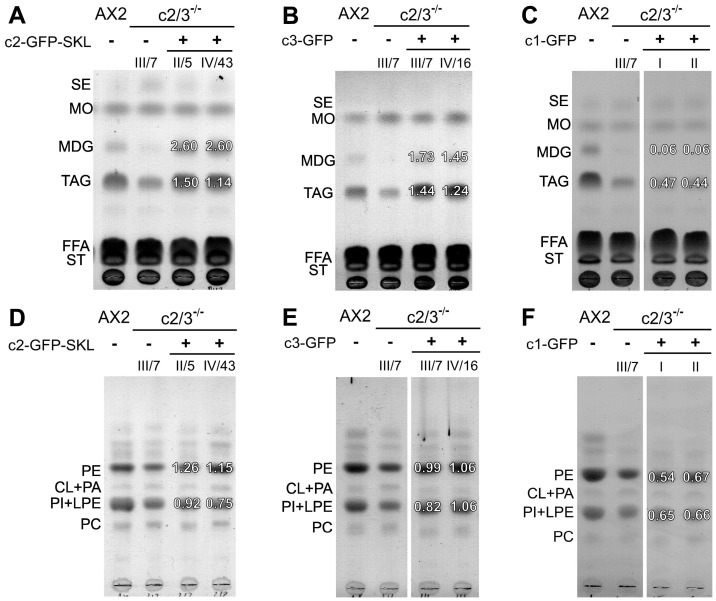
Candidates 2 and 3, but not candidate 1, rescue the double mutant. TLC analyses of neutral lipids (**A**–**C**) and phospholipids (**D**–**F**) of the *c2/3^-/-^* strain III/7 expressing candidates 1 or 3 as hybrids tagged C-terminally with GFP (transformed with plasmids #1415 and #1520, respectively; compare to Figure 2B,G). Candidate 2 was expressed with a GFP-SKL tag at the C-terminus (plasmid #1546, see also Figure 2E). While candidate 1 (c1-GFP) fails to rescue the levels of neutral lipids (**C**) and phospholipids (**F**) of strains I and II, labelled with +, both c2-GFP-SKL (clones II/5 and IV/43, panels (**A**,**D**)) and c3-GFP (lines III/7 and IV/16 in (**B**,**E**)) restore close to wild-type levels of phospholipids and exceed amounts of neutral lipids in the double knockout background. Lipids were analyzed as explained in the legend to Figure 3.

Next, the *c2/3^-^^/^^-^* double knockout background was used to express GFP-tagged versions of each enzyme in an attempt to rescue the defects in lipid synthesis. Expression of candidate 2-GFP-SKL restored the lipid profile seen in the *c3^-^* single knockout, with doubled ether lipids (MDG) and wild-type-like levels of ester lipids (TAGs) (Figure 6A, compare to Figure 4D). In contrast, expression of candidate 3-GFP in the double mutant resulted in slightly elevated levels of both ether and ester lipids (Figure 6B). The levels and patterns of PE and PI phospholipids were rescued to levels close to those of the wild type (Figure 6D,E). Taken together, this confirms the redundancy of candidates 2 and 3 in ether lipid synthesis. Supporting our decision to exclude candidate 1, its expression in the *c2/3^-^^/^^-^* double knockout did not alter the phenotype (Figure 6C,F), indicating that this protein is not a functional ADHAPR.

**Figure 7:**
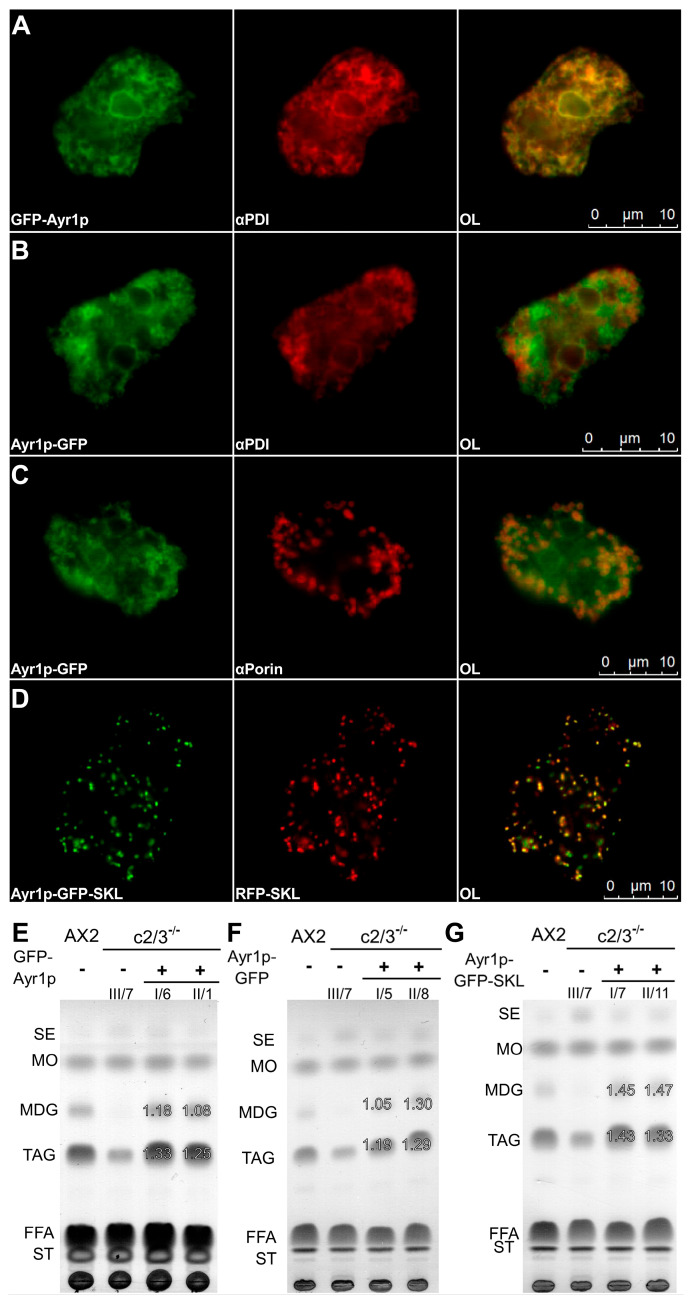
Yeast Ayr1p expression complements the *Dictyostelium c2/3^-/-^* mutant. Three variants of the yeast ADHAPR enzyme Ayr1p were localized in the wild-type background by fluorescence microscopy. GFP-Ayr1p (encoded by plasmid #1584) and the reverse construct Ayr1p-GFP (expressed from plasmid #1585, both in green) coincided with the staining for the PDI protein in the ER (**A**,**B**), but the C-terminally tagged version also bound to mitochondria (identified by porin antibodies in panel (**C**), red). By adding GFP-SKL to its C-terminal end (realized in plasmid #1586), Ayr1p could be forced into peroxisomes, as indicated in panel (**D**) by co-expressing RFP-SKL (red). Otherwise, images were generated as in Figure 2. All three Ayr1p-based constructs were expressed in *c2/3^-/-^* and analyzed for their ability to influence neutral lipid synthesis by TLC (**E**–**G**). Two clones of each construct (GFP-Ayr1p, I/6 and II/1; Ayr1p-GFP, I/5 and II/8; Ayr1-GFP-SKL, I/7 and II/11) independently showed MDG and TAG values slightly exceeding those in wild-type cells (AX2). Lipids were analyzed as explained in the legend to Figure 3.

Finally, we revisited the question of evolutionary conservation of the ADHAPR enzymes by expressing the yeast ortholog Ayr1p (see Figure 1) in the *c2/3^-^^/^^-^* double knockout. GFP-Ayr1p localized to the endoplasmic reticulum (Figure 7A), whereas the reverse-ordered Ayr1p-GFP construct exhibited a dual localization pattern, localizing both to the ER (Figure 7B) and to mitochondria (Figure 7C). This pattern corresponds to the localization of the endogenous Ayr1p in *Saccharomyces cerevisiae* [33,39,40,41]. To see whether this targeting information would override a peroxisomal signal, we produced a hybrid protein Ayr1p-GFP-SKL, which instead distributed entirely to the peroxisomes (Figure 7D). Notably, each of these constructs was able to restore the synthesis of MDG and TAG to about or even above wild-type levels (Figure 7E–G), indicating that the yeast enzyme is fully functional in *Dictyostelium* and candidates 2 and 3 represent true ADHAPR enzymes.

## 4. Discussion

An in silico search in the *Dictyostelium* genome using mammalian ADHAPR as a query sequence has revealed three promising candidates. The most closely related, candidate 1, did not show the expected localization (Figure 1A,B). Moreover, it also failed to rescue a mutant totally devoid of ADHAPR activity (Figure 6C,F). However, lack of a contribution to lipid synthesis pathways does not exclude the annotation of candidate 1 as an enzyme orthologous to *E. coli* ydfG and *S. cerevisiae* TMA29. These proteins are proposed to have NADP^+^-dependent L-serine dehydrogenase activity (http://dictybase.org/gene/DDB_G0282357/feature/DDB0214951, accessed on 12 February 2026), which has not, however, been supported by any previous experiments.

A double knockout of candidates 2 and 3 totally abolished the synthesis of storage ether lipids (MDG in Figure 5D) and also affected phospholipid levels (Figure 5C). The notion that these proteins are bona fide ADHAPR enzymes is strongly supported by the ability of the yeast enzyme to rescue the mutant phenotypes fully (Figure 7E–G). In the double mutant, membrane lipid composition showed drastically reduced PE levels (Figure 5C). PE is known to be the main membrane lipid in *Dictyostelium*, constituting roughly 70% of the total, with 75% thereof being in the ether form [14]. The remaining lipids at this position in the TLC are likely ester-type PE, potentially even increased in amount to compensate for the loss of ether species. PI and lyso-PE are about equal in amount in *Dictyostelium* [14], and it is a limitation of our study that they comigrate on the TLC. The corresponding band is clearly reduced in the double knockout of candidates 2 and 3 (Figure 5C), but we assume that it reflects mainly the loss of ether-PI, which is at 90% of the main form in *Dictyostelium* [14,42]. By their sheer prevalence, ether lipids are expected to be important for cell physiology. Their lack explains an increased generation time in the double mutant (36% above wt). A comparable increase is observed when FARAT, an enzyme that acts upstream of ADHAPR in ether lipid synthesis, is absent (generation time 21% above wt, [11]). As expected, a mutation of FARAT also results in a complete absence of ether-type storage lipids [11]. From these two examples, one can deduce that the cellular metabolism bears enough flexibility to cope with the loss of ether lipids.

Looking more closely, candidates 2 and 3 fulfil quite different tasks according to their localization. Candidate 2 is a peroxisomal enzyme (Figure 2C), and its distribution is mediated by a C-terminal SKL motif (Figure 1B), which constitutes a type 1 peroxisomal targeting signal (PTS1). The human ADHAPR Dhrs7b also localizes to peroxisomes [37,38], but uses a (membrane-) mPTS, i.e., a Pex 19 binding site, which could be assigned to the first 16 amino acids, for targeting instead [34,38]. Interestingly, Dhrs7b also appears in the endoplasmic reticulum in mammalian cells [37,38], dependent on an N-terminal transmembrane domain (position 18 to 38 in Figure 1B) [38,43]. *Dictyostelium* candidate 3 (but not candidate 2) features a signal with similar overall properties (transmembrane domain from position 19 to 39, Figure 1B), and both of its GFP fusion variants indeed accumulate in the ER (Figure 2F,G). From an evolutionary point of view, having two gene products with different subcellular distributions, like in *Dictyostelium*, may reflect the ancestral situation. Since no protein initially carries two competing targeting signals, the ancestor of mammalian Dhrs7b likely possessed a single targeting signal and subsequently acquired a second one, resulting in dual localization. If two functions are fulfilled by a single gene, its sisters Dhrs7(a) and Dhrs7c would then be free to evolve for other tasks. While Dhrs7(a) has been identified as a 3α/20β-hydroxysteroid dehydrogenase [44], regulation of Ca^++^ influx has been attributed to Dhrs7c [45]. On the other hand, interference with Dhrs7b’s function reduces ether lipid levels by only 50% [46,47,48], indicating that a second pathway exists in mammals, which may depend on an as yet unidentified short-chain dehydrogenase.

As far as the function of the *Dictyostelium* ADHAPR candidates is concerned, this discussion is confined to the effect on neutral lipid synthesis, where biochemical effects can be unambiguously assigned without perturbation by compensatory effects and comigrating phospholipids. Both candidates 2 and 3 obviously make a significant contribution to ether lipid synthesis because a drastic phenotype is only seen in a double mutant.

**Figure 8:**
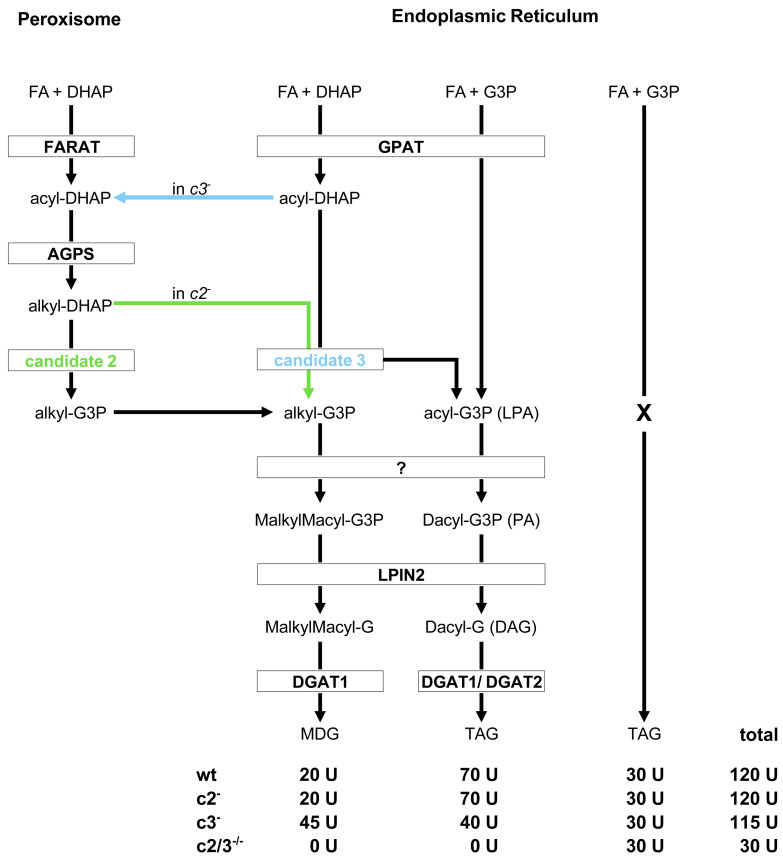
Pathways of neutral lipid synthesis. The processes taking place in the peroxisome or the endoplasmic reticulum are arranged in columns, with the enzymes printed in bold and abbreviated as explained in the text. A question mark (?) designates enzymes not yet identified in *Dictyostelium*. The letter X refers to an entire pathway of TAG synthesis that is as yet unexplained. For substrates and products, the systematic names are used, and common alternative abbreviations are shown in brackets (LPA, lysophosphatidic acid; PA, phosphatidic acid; DAG, diacyl-glycerol). FA signifies fatty acids, and acyl (ester-bound) and alkyl (ether-bound) chains are denoted, with M (mono) or D (di) indicating the chain number on DHAP- or G3P-derived backbones. Black arrows symbolize the flow of lipid compounds in wild-type cells, and coloured arrows visualize alternative routes that are taken in candidate 2 or candidate 3 knockout mutants. The table at the bottom shows the quantitative changes occurring in neutral lipid levels of the single and double ADHAPR candidate mutants as compared to the wild type. We have deliberately decided to show lipid amounts in units (U) here, to explicitly reflect that MDG (20 U) is typically at one-fifth of TAG (a total of 100 U). For TAG values, 1 U therefore equals 1%. Please note that in a candidate 3 mutant, MDG levels increase to about 220% (Figure 4D), equivalent to 45 U. Thus, the mutant has gained 25 U. This, however, occurs at the expense of TAG (dropping by roughly 30%, equaling 30 U) through the pathway shown above by the light blue arrow, while the total neutral lipid sum (total) remains close to the typical value of 120 U. In *c2^-^* cells, the candidate 3 pathway (light green arrow) can fully complement the loss of candidate 2 activity, and amounts of neutral lipids remain unaltered (at 20 U MDG + 100 U TAG) as compared to the wild type. Thus, the coloured arrows reflect the flexible cooperation between peroxisomes and the ER in lipid synthesis of *Dictyostelium*. In a *c2/3* double mutant, only pathway “X” is in operation, leading to a residual amount of 30 U TAG.

Candidate 2 utilizes a substrate, alkyl-DHAP, that is manufactured by the sequential action of FARAT and AGPS in the peroxisome and then reduces the carbonyl group to a hydroxyl moiety, yielding alkyl-glycerol-3-phosphate (alkyl-G3P, see Figure 8) as a product. In a candidate 2 mutant, the substrate should accumulate, but it is instead transported to the ER, where candidate 3 performs the reduction instead of the missing candidate 2. Next, an as yet unknown acyltransferase adds a fatty acid to the sn2 position, and the common enzymes LPIN2 and DGAT1 complete the synthesis of MDG (Figure 8). Thus, no phenotypic alteration is seen in a candidate 2 mutant (Figure 3D).

Being an ER-based enzyme, candidate 3 relies on the substrate that is available locally. GPATs are known to promiscuously use DHAP and G3P as platforms for acylation in mammals and yeast [49,50], yielding acyl-DHAP as well as acyl-G3P. Candidate 3 reduces acyl-DHAP to acyl-G3P, increasing the pool of this product indirectly (see Figure 8). Again, after a second acylation, LPIN2 and DGAT1 or DGAT2 would act sequentially to produce the storage fat TAG. Conceivably, in a candidate 3 mutant, the pool of acyl-G3P would be lower, and less TAG would be produced (Figure 4D and Figure 8). As a second consequence of candidate 3 loss, acyl-DHAP should accumulate, but it is conveyed to the peroxisome instead and further processed by AGPS, yielding more alkyl-DHAP than in the wild type. Enzymatic reduction by peroxisomal candidate 2 to alkyl-G3P thus finally increases the amount of MDG (Figure 4D and Figure 8).

We assume that the flexible flow of metabolites between the ER and peroxisomes occurs via organelle contacts. Close membrane apposition either depends on protein-to-lipid binding [51] or a pair of tail-anchored protein interactions [52,53]. Phospholipids flowing from the ER to peroxisomes are needed for membrane expansion to allow for their subsequent division [53], while cholesterol uses the reverse direction from the peroxisome to the ER [51]. Interestingly, loss of one of the tethering proteins, ACBD5, results in a significant reduction in ether lipid content in siRNA-treated cell lines [53] and human patients [54].

Taken together, *Dictyostelium* cells contain two enzymes with ADHAPR function. Candidate 2 is responsible for the production of ether lipids based on the initial acylation of DHAP in peroxisomes. Candidate 3 channels flux into TAG production originating from DHAP acylation by GPAT in the ER. At the same time and location, GPAT also efficiently acylates G3P (Figure 8). The double mutant of candidates 2 and 3 thus lacks MDG altogether and loses some TAG, but interestingly, residual amounts of TAG are still produced (Figure 5D). Although purely inferential in nature, this observation corresponds well to the finding that genetic deletion of GPAT does not block TAG synthesis fully, but leaves a fraction of 30% unaffected [11]; see path “X” in Figure 8. We conclude from this finding that further research into this as yet unknown path of TAG synthesis in *Dictyostelium* is required.

## Data Availability

The original contributions presented in this study are included in the article/Supplementary Materials. Further inquiries can be directed to the corresponding author.

## Supplementary Materials

The following supporting information can be downloaded at: https://www.mdpi.com/article/10.3390/cells15111025/s1, Table S1: Plasmid list with generation strategy and purpose; Table S2: Primer sequences.

## Abbreviations

The following abbreviations are used in this manuscript:

ADHAPR	alkyl-dihydroxyacetone-phosphate reductase
AGPS	alkylglycerone-phosphate synthase
AT	acyl-transferase
bp	base pairs
BSr	blasticidin resistance
c/c 1/2/3	candidate 1/2/3
CL	cardiolipin
D	di-
DAG	diacyl-glycerol
DGAT	diacyl-glycerol acyl-transferase
DHAP	dihydroxyacetone-3-phosphate = glycerone-3-phosphate
DHAPAT	dihydroxyacetone-3-phosphate acyl-transferase
E	exon
ER	endoplasmic reticulum
FA	fatty acid
FAR	fatty acid reductase
FARAT	fatty acid reductase and acyl-transferase
FcsA	fatty acyl-coenzyme A synthase
FFA	free fatty acid
G	glycerol
G3P	glycerol-3-phosphate
GFP	green fluorescent protein
GPAT	glycerol-3-phosphate acyltransferase
HPTLC	high-performance TLC
IgG	immunoglobulin G
kb	kilo base pairs
LD	lipid droplet
LPA	lysophosphatidic acid
LPE	lyso-phosphatidylethanolamine
Lpin2	lipin 2
M	mono-
MDG	monoalkyl-diacyl-glycerol
MO	methyl-oleate
OL	overlay
PA	phosphatidic acid
PC	phosphatidylcholine
PDI	protein disulfide isomerase
PE	phosphatidylethanolamine
PI	phosphatidylinositol
PTS1	peroxisomal targeting signal type 1 (the amino acid sequence SKL, serine, lysine, leucine)
RFP	red fluorescent protein
SDR	short-chain dehydrogenases/reductase
SE	steryl ester
SKL	see PTS1
ST	sterol
TAG	triacylglycerol
TLC	thin-layer chromatography
U	unit
