# Supplementary material for: Flexible Cooperation Between Peroxisomes and the Endoplasmic Reticulum During Lipid Synthesis of Dictyostelium
Source: Cells. 2026 Jun 2;15(11):1025. doi: 10.3390/cells15111025 (PMC13256971; doi:10.3390/cells15111025)
Supplement: Supplementary file 1 [file cells-15-01025-s001.zip › cells-4304773-supplementary.pdf]

cells

Article

Flexible Cooperation Between Peroxisomes and the Endoplasmic Reticulum During Lipid Synthesis of *Dictyostelium*

Dina Sofia da Silva Telinhos and Markus Maniak *

Zellbiologie, Universität Kassel, Heinrich-Plett-Str. 40, 34132 Kassel, Germany; sofia.telinhos@uni-kassel.de
* Correspondence: maniak@uni-kassel.de

Supplementary Tables

Table S1. Plasmid list with generation strategy and purpose. E: expression, I: intermediate plasmid, KO: knockout

	name		tag	purpose	reference
	pGEM-T easy				Promega GmbH
	pJet1.2				Thermo Fisher Scientific™ Inc.
#393	pLRBLP				[23]
#777	pDneo2a-GFP		GFP		[22]
#800	pDM317		GFP		[21]
#1001	pTX-NLS-CRE			E	[24]
#1035	pDM323		GFP		[21]
#1271	pDneo2a-GFP-SKL		GFP-SKL		[11]
**ID**	**name**	**backbone**	**tag**	**purpose**	**strategy**
#1291	pGEM-T easy BamH I 2651 w/o stop Spe I	pGEM-T easy		I	PCR with primers 1129 & 1130 (gDNA), TA cloning in pGEM-T easy
#1295	pGEM-T easy 2651 + BS^r cassette	#1291		KO	BS^r cassette (#393, Sma I) in #1291 (Smi I)<br>linearized for electroporation with EcoR I and Bgl I
#1400	pJet 1.2 Bgl II 4951 w stop Spe I	pJet1.2		I	PCR with primers 1246 & 1248 (cDNA), blunt in pJet1.2
#1401	pJet 1.2 Bgl II 4951 w/o stop Spe I	pJet1.2		I	PCR with primers 1246 & 1247 (cDNA), blunt in pJet1.2
#1414	pDM317 4951	#800	GFP	E	DDB0214951 (#1400, Bgl II & Spe I) in #800 (Bgl II & Spe I)
#1415	pDM323 4951	#1035	GFP	E	DDB0214951 (#1401, Bgl II&Spe I) in #1035 (Bgl II&Spe I)
#1458	pJet 1.2 Bgl II 2650 w stop Spe I	pJet1.2		I	PCR with primers 1307 & 1309 (gDNA), blunt in pJet1.2
#1471	pJet 1.2 Bgl II 2650 EcoRV (middle) Spe I	#1458		I	Mutagenesis PCR with primers 1328 & 1329 on plasmid #1458

#1474	pJet 1.2 2650+BS^r cassette	#1471		KO	BS^r cassette (#393, Sma I) in #1471 (Sma I) linearized for electroporation with Bgl I, Not I, and EcoR I
#1517	pJet PstI 2650 BamHI	pJet1.2		I	PCR with primers 1383 & 1380 (gDNA), blunt in pJet1.2
#1518	pJet SalI 2650 XhoI	pJet1.2		I	PCR with primers 1381 & 1382 (gDNA), blunt in pJet1.2
#1519	pDneo2A GFP- 2650	#777	GFP	E	DDB0302650 (#1518, Sal I & Xho I) in #777 (Sal I & Xho I)
#1520	pDneo2A 2650-GFP	#777	GFP	E	DDB0302650 (#1517, Pst I & BamH I) in #777 (Pst I & BamH I)
#1536	pJet Pst I 2651 BamH I	pJet1.2		I	PCR with primers 1394 & 1396 (gDNA), blunt in pJet1.2
#1537	pJet Sal I 2651 Xho I	pJet1.2		I	PCR with primers 1395 & 1397 (gDNA), blunt in pJet1.2
#1545	pDneo2A 2651-GFP	#777	GFP	E	DDB0302651 (#1536, Pst I & BamH I) in #777 (Pst I & BamH I)
#1546	pDneo2A 2651-GFP-SKL	#1271	GFP-SKL	E	DDB0302651 (#1536, Pst I/ BamH I) in #1271 (Pst I/ BamH I)
#1547	pDneo2A GFP-2651	#777	GFP	E	DDB0302651 (#1537, Sal I/ Xho I) in #777 (Sal I/ Xho I)
#1582	pJet Sal I ayr1 w stop Xho I	pJet1.2		I	PCR with primers 1412 & 1414 (gDNA yeast), blunt in pJet1.2
#1583	pJet SdaI ayr1 w/o stop BamHI	pJet1.2		I	PCR with primers 1411 & 1413 (gDNA yeast), blunt in pJet1.2
#1584	pDneo2A GFP-Ayr1p	#777	GFP	E	AYR1 (#1582, Sal I/Xho I) in #777 Sal I/Xho I
#1585	pDneo2A Ayr1p-GFP	#777	GFP	E	AYR (#1583, Sda I/BamH I) in #777 Pst I/BamH I
#1586	pDneo2A Ayr1p-GFP-SKL	#1271	GFP-SKL	E	AYR (#1583, Sda I/BamH I) in #1271 Pst I/BamH I

Table S2. Primer sequences. Purpose abbreviated with: C: cloning, CO: control in multiplex, M: mutagenesis, S: sequencing, V: verification

ID	name	purpose	sequence (5′–3′)
871	loxP 3pr	V	ATAACTTCGTATAATGTATGCTATAC
1090	BS^r up		CTGTCGCTACTTCTACTAATTCTAGATC
1091	BS^r down		GAGTTGATTTCAGACTATGCACC
1129	BamHI 2651 fw	C	GGATCCAAAATGACAACAATAACACCATGTTTTATTATAA
1130	2651 XbaI rv w/o stop	C	TCTAGACAATTTTGACTTTAAAGTATTATTATTATTATTATTTGAATTTGAGTTTG
1135	2651 5′UTR	V	CCCCATGTTTTGAATTTAATGC
1139	2651 3′UTR	V	CAAAGGGTGAAGTGTTATGG
1182	DDB0305607 fw	CO	GGATCCATGTCAAATACTTCAAGTGATAATTATAA
1246	BglII 4951 fw	C	AGATCTAAAATGTTTAGAAAAATCTCAAACAAAATTG
1247	4951 rv w/o stop SpeI	C	ACTAGTTTGTTCTCTATAAATATCTCTAGTTGATGC
1248	4951 rv with stop SpeI	C	ACTAGTTTATTGTTCTCTATAAATATCTCTAGTTGATGC
1307	2650 rev with Stop SpeI	C	ACTAGTTTATTTTTTAACAGATGAACTTGCAAATTTATGAG
1309	BglII 2650 fw	C	AGATCTAAAATGTTTTTTTATAAAATAATTTATTTTATTGGTTTTCC
1328	2560 SDM EcoRV for BS^r rv	M	GGTGATTTAACTGATATCTTAATTGATGATTGC
1329	2650 SDM EcoRV for BS^r	M	GCAATCATCAATTAAGATATCAGTTAAATCACC
1337	2650 5′ UTR	V	GGACATTATTCTTAATTGGTTATTGC
1338	2650 3′ UTR	V	GGTGTTTATGGTAAATTCTCACC
1380	2650 w/o stop BamHI rv	C	GGATCCTTTTTTAACAGATGAACTTGCAAATTTATGAG
1381	SalI+A 2650	C	GTCGACAATGTTTTTTTATAAAATAATTTATTTTATTGGTTTTCC
1382	2650 with stop XhoI rv	C	CTCGAGTTATTTTTTAACAGATGAACTTGCAAATTTATGAG

1383	PstI+AA+2650	C	CTGCAGAAAATGTTTTTTTATAAAATAATTTATTTTATTGGTTTTCC
1394	PstI AA 2651 fw	C	CTGCAGAAAATGACAACAATAACACCATGTTTTATTATAAC
1395	SalI+A 2651	C	GTCGACAATGACAACAATAACACCATGTTTTATTATAAC
1396	2651 rv w/o stop BamHI	C	GGATCCCAATTTTGACTTTAAAGTATTATTATTATTATTATTTGAATTTGA
1397	2651 rev mST XhoI in #777	C	CTCGAGTTACAATTTTGACTTTAAAGTATTATTATTATTATTATTTGAATT
1411	SdaI +AA+AYR1 fw	C	CCTGCAGGAAAATGTCGGAGTTACAGTCACAACC
1412	SalI+A AYR1 fw	C	GTCGACAATGTCGGAGTTACAGTCACAACC
1413	AYR1 w/o stop BamHI rv	C	GGATCCATCGTCCTTATTCTTCTGTTTCG
1414	AYR1 with stop XhoI rv	C	CTCGAGCTAATCGTCCTTATTCTTCTGTTTCG
1416	DDB0305607 middle rv	CO	GATATTGTGCTGGTAAATCTGC
